# The methodology for assessing smoking-attributed mortality based on All Causes of Death Surveillance in Tianjin, China, 2010–2015

**DOI:** 10.18332/tid/116970

**Published:** 2020-03-23

**Authors:** Wei Li, Dezheng Wang, Hui Zhang, Ying Zhang, Wenlong Zheng, Xiaodan Xue, Wenda Shen, Freddy Sitas, Guohong Jiang

**Affiliations:** 1 Department of Non-communicable Disease Control and Prevention, Tianjin Centers for Disease Control and Prevention, Tianjin, China; 2 School of Public Health, Tianjin Medical University, Tianjin, China; 3 Centre for Primary Health Care and Equity, School of Public Health and Community Medicine, University of New South Wales Sydney, Sydney, Australia; 4 Menzies Centre for Health Policy, University of Sydney, Sydney, Australia; 5 School of Public Health, University of Sydney, Sydney, Australia

**Keywords:** smoking-attributed mortality, mortality surveillance, death certificate, relative risk

## Abstract

**INTRODUCTION:**

The All Causes of Death Surveillance (ACDS) system was used to measure smoking-attributed mortality by inserting questions on smoking on death certificates. Smoking status information of the deceased has been routinely collected in death certificates since 2010. We describe a death registry-based case-control study using smoking and cause-of-death data for the period 2010–15.

**METHODS:**

From 2010, three questions about the smoking status of the deceased were inserted in a revised death certificate: 1) Smoking status (current smoker, quit smoking, never smoker); 2) Number of cigarettes per day smoked; and 3) Number of years of smoking. A data-accuracy survey of 1788 telephone interviews of the family of the deceased was also conducted. Smoking habits (current/ex-smoker vs non-smoker) were compared in study cases (persons who died of lung cancer and other diseases known to be caused by smoking) and the controls (never smokers). Multivariate logistic regression analysis was conducted to estimate relative risks, RR (odds ratios) for smoking-attributed mortality, for lung cancer and all causes of death related to smoking, adjusted for 5-year interval age groups, education, marital status, and year of death.

**RESULTS:**

During the study period (2010–15), the annual crude death reporting rates ranged from 6.5‰ to 7.0‰. The reporting rates of smoking status, smoking history and the number of cigarettes smoked daily were 95.5%, 98.6% and 98.6%, respectively. Compared to never smokers, the RR of ever smoking in males was 1.38 (95% CI: 1.33–1.43) for all causes of smoking-related deaths and 3.07 (95% CI: 2.91–3.24) for lung cancer, while in females the values were 1.46 (95% CI: 1.39–1.54) for all causes of smoking-related deaths and 4.07 (95% CI: 3.81–4.35) for lung cancer. The results in Tianjin are in accord with published results from previous studies.

**CONCLUSIONS:**

Levels and trends in smoking attributed mortality can be measured at low cost by using the stable, complete and effective ACDS system in Tianjin.

## INTRODUCTION

As the world’s largest tobacco consumer, China has about 1.2 million people dying from smoking-related causes each year^1^. The majority of adult males (52.1%) and a small proportion (2.7%) of adult females in China are current smokers, while the rate is respectively 24.3% and 2.9% in India, and 30.1% and 7.9% in Japan. Meanwhile, in the US, smoking patterns differ, being lower in males (17.4%) and higher in females (13.0%) compared to the above countries^2^. Introduction of tobacco control policies, especially the implementation of smoke-free laws, has been slow; one of the main reasons being a lack of awareness of the harms of tobacco, both by governments and by the public. While studies are emerging in China, the proportion of deaths attributed to smoking can vary significantly from place to place^3^. At present, much of the evidence has been derived from large cohort studies of smoking attributed mortality^4–9^. However, in most areas of China, it is difficult to carry out large cohort studies due to resource limitations. As a practical alternative, Tianjin Centers for Disease Control and Prevention (CDC) used existing resources from the ACDS system to collect smoking information of the deceased, following a method reported previously^3^, to provide more accurate and comprehensive data for monitoring the effect of tobacco control.

**Figure 1:**
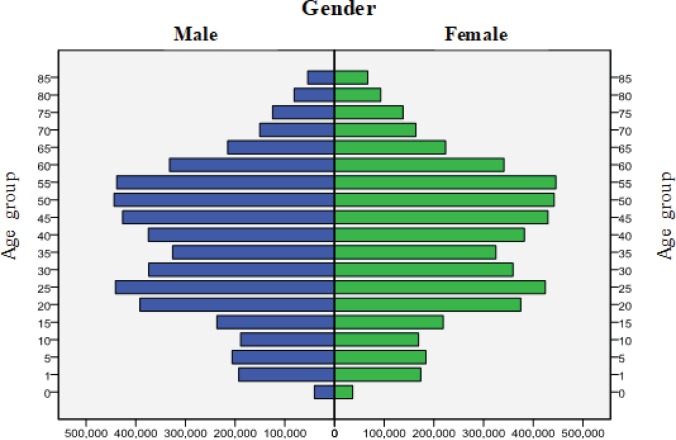
Population pyramid in Tianjin

Tianjin is a port city in North–East China close to Beijing (the capital of China) and has a population of about 10 million household residents in 16 districts; the GDP per capita was ranked third in China after Shanghai and Beijing^10^. About 62% of the residential population in Tianjin lives in urban areas. Figure 1 shows the population-pyramid of Tianjin (see Supplementary file, Map of Tianjin), which, as in most cities of China^11^, shows slow fertility rates and an emerging older population.

At the end of 2009, Tianjin CDC inserted three questions about the smoking habits of the deceased on the revised death certificate. By the end of 2015, there were registered 396113 deaths that contained information on smoking to ascertain its relation to 105 different causes of death^12,13^. Such data and evidence was used to promote the formulation of the smoke-free laws in Tianjin, to meet the requirements of the WHO Framework Convention on Tobacco Control^14^. In the future, these data will be continually used to monitor the disease burden of smoking and the health benefits of cessation.

This report describes how the All Causes of Death Surveillance (ACDS) system was utilised to measure smoking attributed mortality in Tianjin and provides some preliminary results to illustrate its usefulness.

## METHODS

### ACDS system in Tianjin

**Figure 2:**
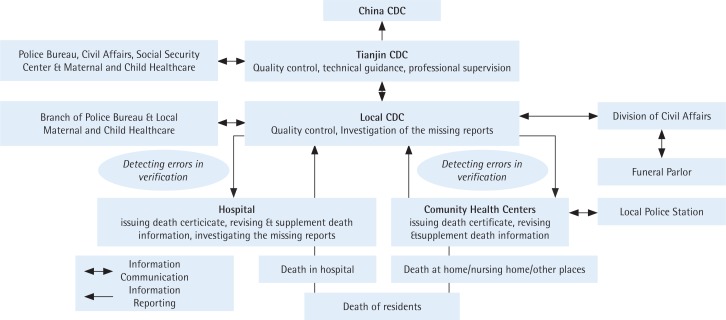
Network of the All Causes of Death Surveillance system

The ACDS system in Tianjin was developed in 1984 and covers the whole household registered population in Tianjin. Through this system, all causes of death should be reported, and all death certificates entered into the database must be completed by physicians in hospitals or community clinical centres. Physicians determine the underlying cause of death of the deceased, based on ICD-10 rules, according to the clinical manifestations of the deceased, combined with imaging examination, laboratory tests and medical records. The process of death registration and the further developments of the death certification system were described in the Jiang et al.^12^ study. Figure 2 shows the procedure and network of the ACDS system.

### Smoking information collection by the ACDS system

Each death certificate includes more than 50 data fields, including age, sex, and cause of death, as well as the home address used to classify the area of residence as urban or rural (Supplementary file, Document 1 shows the Chinese and English versions of the death certificate in Tianjin). At the end of 2009, Tianjin CDC inserted three questions about the smoking habits of the deceased on the revised death certificate or death medical questionnaire, which were approved by the TJCDC Ethics Committee. Questions include: 1) Smoking status (current smoker, quit smoking, never smoker), 2) Number of cigarettes smoked per day, and 3) Number of years of smoking. A smoker was defined as someone who smoked continuously for at least 6 months in his/her lifetime. An ex-smoker was defined as someone who stopped smoking at least one year prior to death. Instructions about how to fill the smoking information on the death certificate were issued by the Tianjin Public Health Bureau, which is the administrative department of Tianjin CDC and which issued the regulations for all of the doctors to report the smoking information on the death certificate. In the meantime, specific regular training and supervision was developed for staff in local CDCs and doctors in hospitals. On most occasions, high ranking members of the Tianjin Public Health Bureau were invited to give talks emphasizing the importance of mortality surveillance. As a result of feedback from surveys, TJCDC will develop more targeted quarterly training and supervision.

### Quality control

#### Quality control program application

A computerised quality control program was developed to automatically filter out errors, such as missing smoking information, and obvious logical errors, for example ID number verification error and street/block name error etc. Tianjin CDC sent monthly error reports to local CDCs through the ACDS system. The local CDCs were given the task of providing feedback on errors to the appropriate hospitals, requesting them to investigate, verify and modify any errors within 5 days^15,16^.

#### Under-reporting survey

To enhance the death reporting rate, Tianjin CDC developed an ongoing survey to estimate under-reporting of deaths. For deaths in hospitals, the survey included checking medical records, accounts of hospitalisation registration, outpatient and emergency treatment logs in different clinical departments, for references to a death. For deaths outside hospitals, different methods to estimate under-reporting were carried out in rural and urban districts, respectively. In urban areas a street was chosen from three streets whose crude death rate was similar to the whole district. All of the residents’ death information in the selected street was collected from the community health service center, and compared with information from the ACDS system^17^. In rural districts, capture-recapture methodology was used to compare the death records in the ACDS system with the funeral parlor registration system, in order to identify under-reporting. The concordant and discordant deaths were estimated according to the ID-card number, name, sex, age, and street. Medical institutions routinely report the total number of deaths and the rate of missing reports. Districts with poor reporting rates are referred to the local Public Health Bureau, a local government department, and their performance is noted in their annual performance evaluations. Low reporting rate districts were also reported to TJCDC with the aim to provide specialised technical guidance and supervision to help resolve problems and to monitor improvements.

#### Smoking information

The accuracy of smoking information was checked through a telephone interview in 2015 concerning the data from 2010–15. The sample size of the call-back survey was calculated according to the proportion of missing information on smoking, reported annually^18^. The sample size was calculated from the formula:

$$\text{n}=deff\times u^{2}p(1-p)/\delta^{2}$$

where the level of significance α = 0.05, *u_α_* = 1.96, *p* (estimated accuracy rate) = 0.8, *δ* (tolerance error) = 0.8×10% = 0.08, deff (design effect) = 1.2, giving n=115. Stratified sampling was performed considering urban and rural areas (2 levels), death location (hospital and outside hospital, 2 levels) and smoking status (smoker and non-smoker, 2 levels). Taking into account the stratification levels and response rate (about 80%), total sample size = 115×2×2×2/0.8=1150. All causes of smoking related death include 11 groups of diseases that followed the methods of Sitas et al.^8^ in 10 groups positively (tuberculosis; chronic obstructive pulmonary disease; other respiratory disease; cerebrovascular diseases; ischemic heart disease; other cardiovascular disease; cancer of the lung; cancer of mouth, pharynx, larynx, or esophagus; cancer of stomach, liver, or pancreas; and cancer of cervix or urinary tract, myeloid leukemia) and in 1 group negatively (Parkinson disease, ulcerative colitis, and endometrial cancer).

### Statistical analysis

Multivariable logistic regression analysis was used to estimate smoking-related (independent variable) odds ratios (= relative risk) of death for all smoking-related causes of death and for lung cancer (dependent variable), versus controls in males and females, respectively. These are adjusted for confounding, 5-year interval age group, sex and education (none, primary, higher, do not know), and marital status (never, widowed, divorced, married or living as married, do not know). Smoking related mortality relative risks were compared to results from previous large cohort and case-control studies. All statistical analyses were performed using SPSS 16.0 (SPSS, Chicago, IL)^19^.

## RESULTS

There were more than 70000 deaths reported every year during 2010–15, giving a crude death rate of 6.5–7‰ (Table 1). The completeness rates of smoking status, number of cigarettes smoked, and number of years of smoking were 95.5%, 98.6% and 98.6%, respectively. More than 85% of causes of death were diagnosed by secondary hospitals and above. The proportion diagnosed by tertiary hospitals was more than 65%. In a special investigation from 2009 to 2013, the under-reporting rates of deaths in hospitals for each year were low: 2.7, 1.4, 0.5, 0.8 and 1.2%, respectively, which are relatively low and stable.

**Table 1:** Crude death rate in males and females in 2010–2015, Tianjin, China

*Year*	*Crude death*	*Crude death rate in males (‰)*	*Crude death rate in females (‰)*
2010	6.95	7.63	6.27
2011	6.60	7.29	5.90
2012	6.88	7.62	6.13
2013	7.00	7.74	6.25
2014	7.07	7.87	6.26
2015	7.14	7.91	6.36

### Cause of death reporting and accuracy of smoking information

Table 2 shows the number of registered deaths and rate of missing information, inaccurate diagnosis compared to cause of death coding between 2010–15. The rate of missing smoking information in the death reports declined from 10.95% in 2010 to 0.84% in 2015.

**Table 2:** Percentage of ill-defined codes and missing smoking information in the All Causes of Death Surveillance system in 2010–2015, Tianjin, China

*Year*	*Number of reported deaths*	*Unknown causes of death*	*Cardiovascular disease categories lacking diagnostic meaning*	*Cancer deaths coded as secondary or unspecified*	*Respiratory, heart or liver failure, cardiac arrest*	*Missing rate of smoking status*
2010	70082	1.33	0.99	0.03	0.04	10.95
2011	68272	1.18	0.90	0.03	0.06	6.38
2012	72268	1.25	1.38	0.01	0.06	2.52
2013	73472	1.40	1.51	0.01	0.06	1.71
2014	72843	1.60	1.98	0.04	0.05	1.15
2015	76056	1.83	2.12	0.04	0.03	0.84

### Confirmation of smoking information by telephone interview

A random sample survey of 1788 death reports (see sample size calculation above) that had smoking information was developed through telephone interview by CDC staff and doctors in hospitals. Once missing values were excluded (missing values from death certification by doctors), the kappa statistic between the death certification and telephone interview was 0.75, which suggests excellent agreement (Table 3). The main reasons for the inconsistencies were: information not filled in, transcription errors, and data input errors.

**Table 3:** A random sample survey result comparing smoking status on death certificate with that from a telephone survey in 2015, Tianjin, China

*Smoking status*		*Death certification by doctors*		
		*Ever smoker*	*Never smoker*	*Missing*
Telephone interview by CDC staff	Ever smoker	1058	143	45
	Never smoker	61	524	80
	Missing	1	1	0
Kappa	3×3		0.65	
	2×2 excluding missing		0.75	

### Mortality attributed to smoking in Tianjin compared with other regions or countries

**Figure 3:**
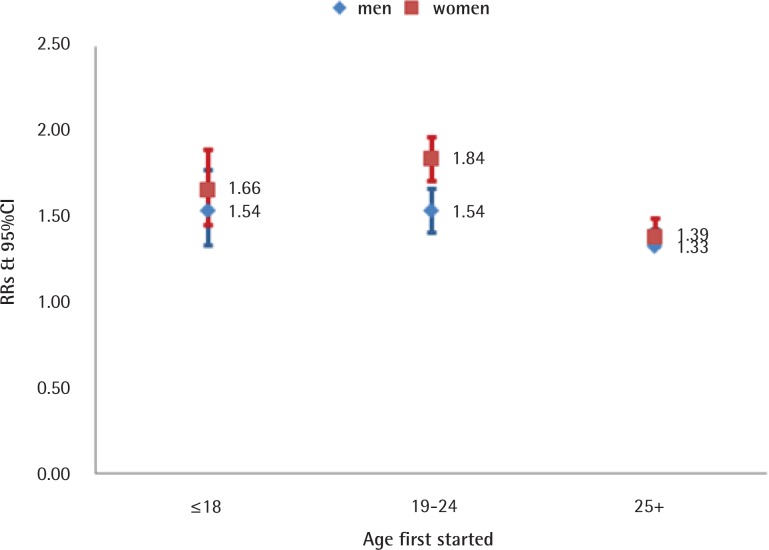
Age started smoking. All cause of death in men and women, aged 35–79 years, ever smokers vs never smokers, 2010–2014

**Figure 4:**
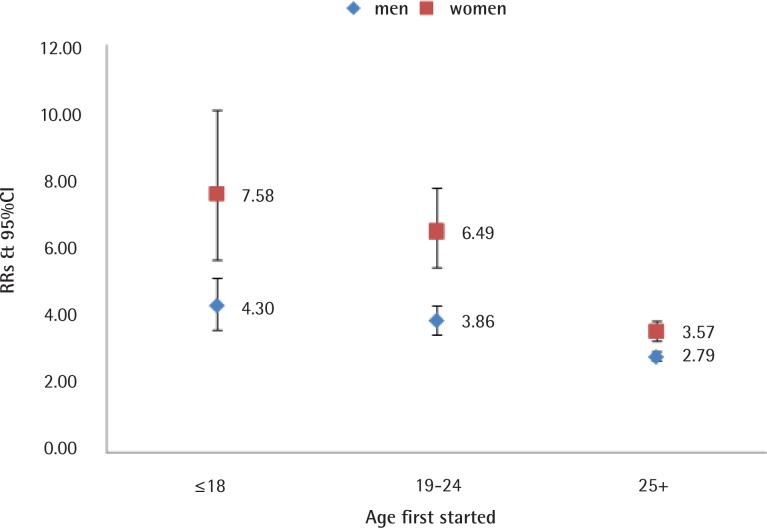
Age started smoking. Lung cancer in men and women, aged 35–79 years, ever smokers vs never smokers, 2010–2014

RRs (adjusted) were elevated in all causes of smoking-related death and lung cancer both in men (All causes of death: RR=1.38, 95% CI: 1.33–1.43; Lung cancer: RR=3.07, 95% CI: 2.91–3.24) and women (All causes of death: RR=1.46, 95% CI: 1.39–1.54; Lung cancer: RR=4.07, 95% CI: 3.81–4.35). Females who started smoking at 18 years or younger had higher RR for lung cancer (RR=7.58, 95% CI: 5.92–10.38) compared to those starting at age 19–24 years (RR=6.49, 95% CI: 5.27–7.82) and ≥25 years (RR=3.57, 95% CI: 3.26–3.93;). Similar, but less pronounced results in relation to starting smoking age were found for men (Figures 3 and 4).

In order to verify the reliability and accuracy of smoking attributed mortality results by the ACDS system, results in men were compared with published results of domestic and overseas well-known studies of smoking-attributable death^1,7–9,20,21^. The relative risks in our analysis were essentially in agreement with other related studies, which were 1.21–2.00 for all causes of death and 2.44–5.17 for lung cancer in men, and 1.23–1.62 for all causes of death and 1.52–4.13 for lung cancer in women (Supplementary file, Figures 1 and 2).

## DISCUSSION

The Tianjin ACDS system was founded in 1984 and covers the whole household registered population in Tianjin of 10 million. Through continuous training, supervision, assessment and update of software technology, Tianjin developed close coordination between public health agencies, like the CDC, and clinics and hospitals. The quality of death reports has been constantly improving. The death report rate has been stable 6.5–7.0‰ from 2005, while the rate of missing reports, the proportion of information missing, coding errors and diagnosis for the cause of death unknown, have been improving year by year. The data quality of death surveillance of Tianjin has been ranked in the leading position in China^22,23^.

In 2009, Tianjin CDC achieved funding from Bloomberg Global Tobacco Control Foundation to carry out the ‘100% smoke-free environment’ project, aiming at promoting smoke-free legislation. In Butin, Tianjin, and in many parts of China, there had been a lack of data about smoking attributed mortality and associated disease burden. Such ongoing disease surveillance initiatives are highly cost-effective, informative and necessary mechanisms to inform stakeholders, legislators and the public by documenting the local harms of tobacco and monitoring progress.

Through effective communication with the hospitals and the different agencies involved in death notification, almost all deaths are reported, the fill-in rate of smoking information is 98%, and the accuracy rate (kappa) is excellent (0.75). We used this information to calculate smoking attributed mortality to assess the risks for all causes and major causes of mortality with smoking, in Tianjin^12^. The results are in accord with published results in domestic and overseas well-known smoking-attributable death cohort studies. This study also provides evidence to promote the promulgation and implementation of the Tianjin Tobacco Control Regulation. This is the first death-registration system in China that has added questions on smoking. Through the accumulation of the data year-by-year, Tianjin will have increasingly large, reliable and representative data about smoking and premature death.

### Limitations

Our study has some limitations. First, the definition of study cases and controls would need further research, because new diseases have been confirmed to be causally associated with smoking, which were defined as a control disease in our study following Sitas et al.^8^. This could have under-estimated the RR of smoking. Second, our ever smokers included former smokers. As most smokers stopped smoking due to illness and few stop by choice in China, including them would have under-estimated the current versus never smoker values of RR. We added another question about quitting-decade on the death certificate from 2015.

## CONCLUSIONS

The study shows that countries or regions can obtain smoking attributable mortality data by utilising their death notification system. Such information will provide important evidence to measure the harms of tobacco and to monitor the benefits of quitting.
