# Supplementary material for: The methodology for assessing smoking-attributed mortality based on All Causes of Death Surveillance in Tianjin, China, 2010–2015
Source: Tob Induc Dis. 2020 Mar 23;18:21. doi: 10.18332/tid/116970 (PMC7132577; doi:10.18332/tid/116970)
Supplement: Supplementary file 1 [file TID-18-21-s1.pdf]

居民死亡医学证明（推断）书

______省(自治区、直辖市)______市(地区、州、盟)______县(区、旗)

行政区划代码□□□□□□　　　　　　　　死亡卡编号：　　　　　　　　编号：2015000001

死者姓名		性别	1 男，0 未知的性别<br>2 女，9 未说明的性别	民族		国家或地区	
有效身份证件类别	1 身份证,2 户口簿,3 护照<br>4 军官证,5 驾驶证<br>6 港澳通行证,7 台湾通行证<br>9 其他法定有效证件	证件号码		年龄		婚姻状况	1 未婚,2 已婚,3 丧偶<br>4 离婚，9 未说明
出生日期	年　月　日	文化程度	1 研究生,2 大学,3 大专<br>4 中专，5 技校，6 高中<br>7 初中及以下	个人身份	11 公务员，12 专业技术人员，17 职员<br>21 企业管理者，24 工人，27 农民，31 学生<br>37 现役军人,51 自由职业者，54 个体经营者<br>70 无业人员,90 其他		
死亡日期	年　月　日<br>时　分	死亡地点	1 医疗卫生机构,2 来院途中,3 家中<br>4 养老服务机构,9 其他场所,0 不详		死亡时是否处于妊娠期或妊娠终止后 42 天内		1 是，2 否
生前工作单位		常住地址	省　市　区(县)　街(乡、镇)　居(村)　号				
户籍地址	省　市　区(县)　街(乡、镇)　居(村)　号						
可联系的家属姓名		联系电话		家属住址或工作单位			

致死的主要疾病诊断	疾病名称（勿填症状体征）	发病至死亡大概间隔时间
I.(a)直接死亡原因		
(b)引起(a)的疾病或情况		
(c)引起(b)的疾病或情况		
(d)引起(c)的疾病或情况		
II.其他疾病诊断（促进死亡，但与导致死亡无关的其他重要情况）		

生前主要疾病最高诊断单位	1 三级医院，2 二级医院，3 乡镇卫生院/社区卫生服务机构，<br>4 村卫生室，9 其他医疗卫生机构，0 未就诊	生前主要疾病最高诊断依据	1 尸检,2 病理,3 手术,4 临床+理化<br>5 临床，6 死后推断，9 不详

备注：吸烟情况：A1)吸烟　A2)戒烟　A3)从不吸烟；　吸烟时每天吸烟支数：B　　支，累计吸烟年限：C　　年
如果死者曾患恶性肿瘤或中枢神经系统良性肿瘤，请填写首次确诊日期：　　　年　　月　　日

住院号		医师签名		医疗卫生 机构盖章	填表日期：　年　月　日
（以下由编码人员填写）根本死亡原因：					ICD 编码：

死亡调查记录

死者生前病史及症状体征：

以上情况属实，被调查者签字

**被调查者姓名**		**与死者关系**		联系电话		**联系地址或工作单位**	
**死因推断**				**调查者签名**		**调查日期**	年　月　日

注：①此表填写范围为在家、养老服务机构、其他场所正常死亡者；②被调查者应为死者近亲或知情人；③调查时应出具以下资料：被调查者有效身份证件，居住地居委会或村委会证明，死者身份证和/或户口簿、生前病史卡.

天津市疾病预防控制中心印制

Residents' Medical Death Certificate

______Provinece(Autonomous region, municipalitiesdirectly under the Central Government)______City(Area, State, Union)______Country(District)

Codes for the administrative divisions □□□□□□　　　　Death card number: No: 2015000001

Name		Sex	1 Male, 0 Unknown<br>2 Female, 9 Unaccounted	Nationality		Country/Area	
Effective Identification	1 Identification card, 2 Household regist, 3 Passport 4 Military ID, 5 Driver's license, 6 Hong Kong and Macao pass, 7 Tai Wan pass, 9 Other legal effective certificates	Identity Number		Age		Marital status	1 Single<br>2 Married<br>3 Widowed<br>4 Divorced<br>9 Unaccounted
Date of birth	___Year___Month___Day	Education	1 postgraduate, 2 college,<br>3 junior college,<br>4 polytechnic school,<br>5 Technical school,<br>6 high school,<br>7 Junior middle school and the following	Type of business	11 civil servant, 12 professionals, 17 office clerk, 21 business manager, 24 worker, 27farmer, 31student, 37 active army, 51 free agent, 54 self-employed, 70 unemployed, 90 other		
Date of Death	___Year___Month___Day<br>___Hour___Minute	Place of Death	1 Hospital, 2 On the way to the hospital, 3 Home 4 Aged Support Agencies, 9 Other, 0 Unknown	If a female, was she pregnant or Within 42 days after termination of pregnancy		1 Yes<br>2 No	
Usual occupation of deceased		Usual residential address of deceased	__Province____City___District (Country)_Street (Town)_ Neighborhood committee (Village)____No.				
Residence address	__Province___City____District (Country)____Street (Town)___Neighborhood committee (Village)_________No.						
Name of family member can be contacted		Tel		Address or work unit of the family member			

Medical certificate of cause of death	Enter the disease, injuries or complications that caused the death. Do not enter the mode of dying, such as cardiac or respiratory arrest, shock, or heart failure. List only one cause on each line.	Approximate interval between onset and death
I.(a)Immediate cause		
(b) Due to a (or as a consequence of a)		
(c) Due to b (or as a consequence of b)		
(d) Due to c (or as a consequence of c)		
II. Other significant conditions contributing to death but not resulting in the underlyding cause given in Part I.		

The highest diagnostic unit	1 Tertiary hospitals, 2 Secondary Hospital,<br>3 Health clinic in town/ Community health serveice center,<br>4 Village health room, 9 Other medical and health institutions,<br>0 Have no treatment	Method of ascertainment of cause of death	1 Autopsy, 2 Pathology, 3 Operation, 4 Clinic & physic-chemical 5 Clinic, 6 Deduced after death, 9 Unknown

Remarks: (1) Smoking status A1 current smoking, A2 quit smoking, A3 never smoked. (2) Number of cigarettes smoked when smoking ____per day (3) Total number of years of smoking ___years

If the deceased had a malignant tumor or benign tumor of the central nervous system, please fill in the date of first diagnosis:____Year Month___Day

Inpatient number		Physician's Signature		Seal of hosipital or community health service center	Date: __Year___Month___Day
(The following is filled by the coding physicians) Immediate cause of death					ICD:

Death survey records

The history of the deceased and the symptoms and signs:<br><br>The above is true, the signature of the investigator

Name of the person being investigated		Relationship to deceased		Tel		Contact address or work department	
Verbal autopsy				Signature of the investigator		Date of Investigation	__Year___Month___Day

Notes：①This form is to be filled out in the form of a normal dead person at home, in the old age service, and in other places.；②The investigator shall be a close relative or partner；③Investigation should provide the following information: the valid identity documents of the respondents, the residence of the neighborhood or village committee to prove that the deceased's identity card and/or residence booklet, living history card.

Tianjin Centers for Disease Control and Prevention Print

© 2020 Li W. et al.
