# Supplementary material for: The methodology for assessing smoking-attributed mortality based on All Causes of Death Surveillance in Tianjin, China, 2010–2015
Source: Tob Induc Dis. 2020 Mar 23;18:21. doi: 10.18332/tid/116970 (PMC7132577; doi:10.18332/tid/116970)
Supplement: Supplementary file 2 [file TID-18-21-s2.pdf]

Figure 1. Smoking attributed mortality in all causes of death and lung cancer in men in Tianjin and other counties/regions

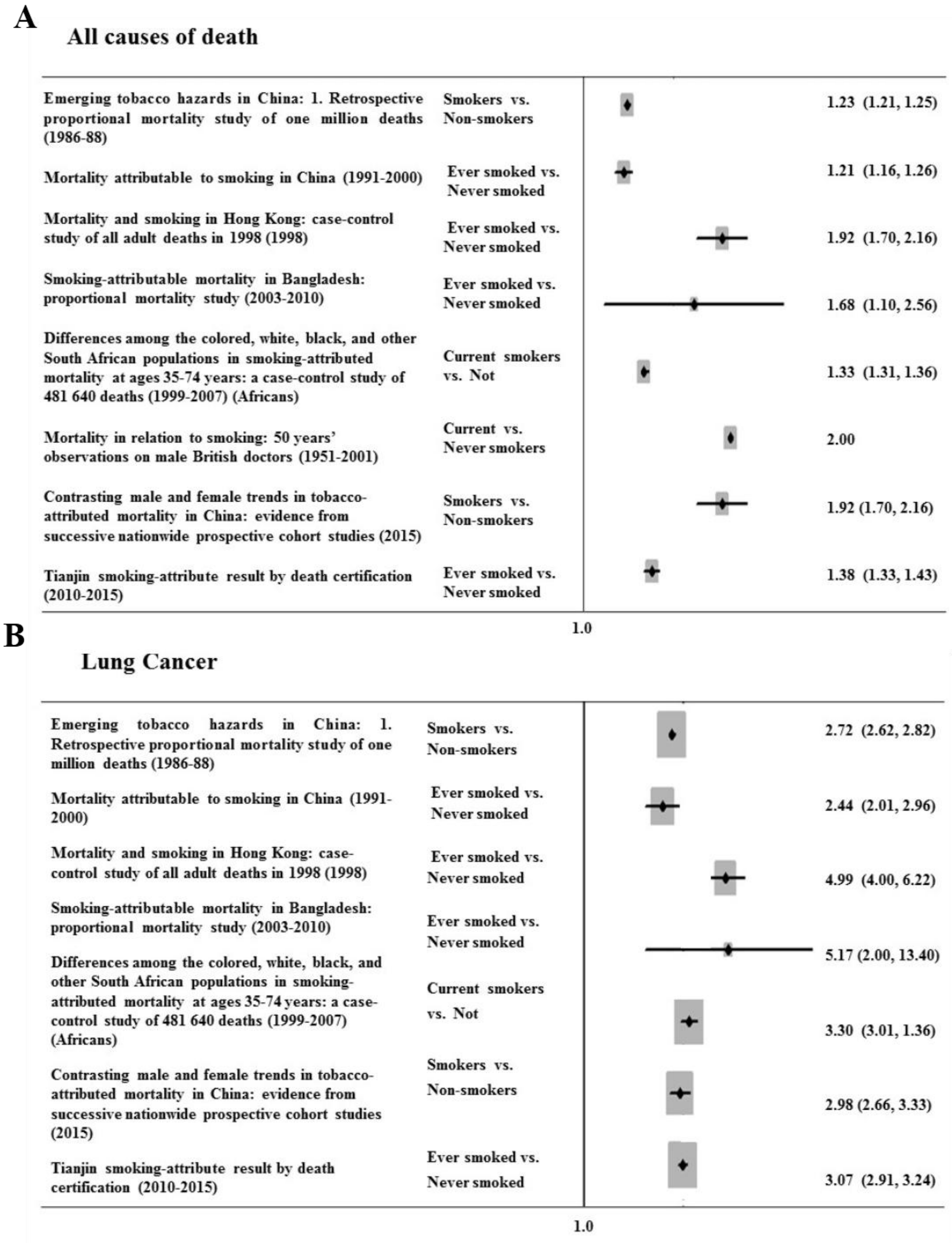

Figure 2. Smoking attributed mortality in all causes of death and lung cancer in in women in Tianjin and other counties/regions

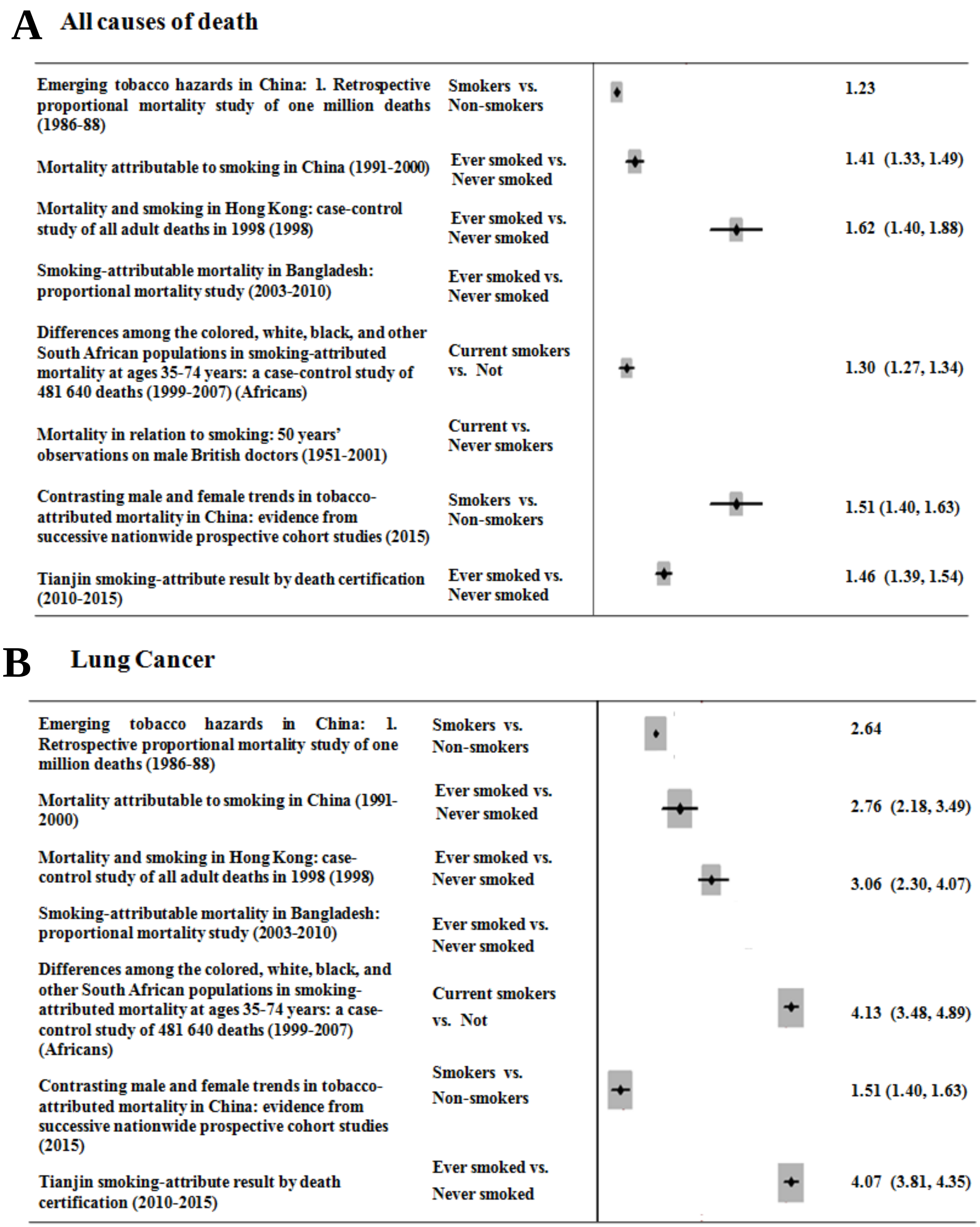

© 2020 Li W. et al.
