# Supplementary figures and images for: The methodology for assessing smoking-attributed mortality based on All Causes of Death Surveillance in Tianjin, China, 2010–2015
Source: Tob Induc Dis. 2020 Mar 23;18:21. doi: 10.18332/tid/116970 (PMC7132577; doi:10.18332/tid/116970)

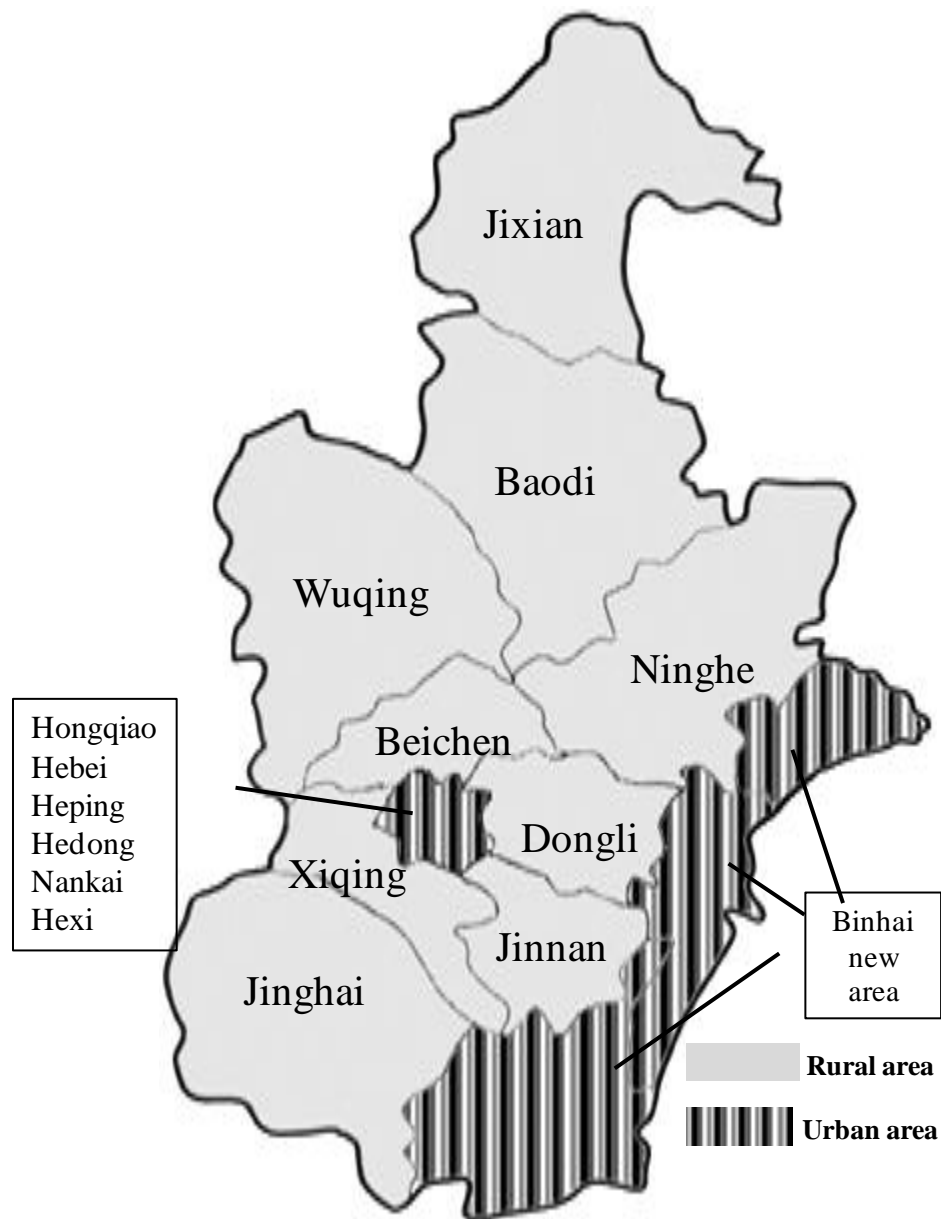

*Map of Tianjin, China*

Supplement: Supplementary file 3 [file TID-18-21-s3.pdf]
